# Short-term survival and safety of apatinib combined with oxaliplatin and S-1 in the conversion therapy of unresectable gastric cancer

**DOI:** 10.1186/s12885-021-08459-3

**Published:** 2021-06-15

**Authors:** Zaisheng Ye, Yi Zeng, Shenghong Wei, Yi Wang, Zhitao Lin, Shu Chen, Zhiwei Wang, Shanshan Chen, Luchuan Chen

**Affiliations:** 1 Department of Gastrointestinal Surgical Oncology, Fujian Cancer Hospital & Fujian Medical University Cancer Hospital, Fuzhou, 350014 Fujian Province China; 2 Department of Fujian Provincial Key Laboratory of Tumor Biotherapy, Fujian Cancer Hospital & Fujian Medical University Cancer Hospital, Fuzhou, 350014 Fujian Province China

**Keywords:** Unresectable gastric cancer, Conversion therapy, Apatinib, Efficacy, Safety

## Abstract

**Background:**

We conducted a single-arm phase II trial to investigate the short-term efficacy and safety of apatinib combined with oxaliplatin and S-1 in the treatment of unresectable gastric cancer.

**Patients and methods:**

Previously untreated patients with unresectable HER-2-negative advanced gastric cancer were selected. All the patients received six cycles of S-1 and oxaliplatin and five cycles of apatinib, which were administered at intervals of three weeks. The surgery was performed after six cycles of drug treatment. The primary endpoints were radical resection (R0) rate and safety. This study was registered with the China Trial Register, number ChiCTR-ONC-17010430 (01/12/2016–01/12/2022).

**Results:**

A total of 39 patients were enrolled. Efficacy evaluation was feasible for 37 patients. One patient achieved complete response (CR, 2.7%), 26 patients achieved partial response (PR, 70.3%), three patients had stable disease (SD, 8.1%) and seven patients had progressive disease (PD, 18.9%). The objective response rate (ORR) was 73.0% and the disease control rate (DCR) was 81.1%. 22 patients underwent surgery, among which 14 patients underwent radical resection (R0), with a R0 resection rate of 63.6%. The 1-year survival rate of the surgical group (22 patients) was 71.1% and the 2-year survival rate was 41.1%. The median survival time was 21 months. The incidence of adverse events (AEs) was 100%. Leucopenia (65.3%) and granulocytopenia (69.2%) were the most common hematological AEs. The most common non-hematological AEs were fatigue (51.3%) and oral mucositis (35.9%).

**Conclusion:**

Apatinib combined with oxaliplatin and S-1 showed good short-term survival and acceptable safety in the conversion therapy of unresectable gastric cancer.

## Background

In China, unresectable gastric cancer accounts for 10% of the total number of gastric cancer cases [1]. At present, palliative chemotherapy is the main treatment option. The median survival time is 5–12 months, and the 5-year survival rate is about 9.4% [2]. In unresectable gastric cancer, the primary focus of gastric cancer infiltrates into the extraserous or surrounding tissues and organs, and distant metastasis occurs, such as paraaortic, liver, peritoneum, etc. Therefore, radical resection is difficult from the perspective of surgical technology and oncology. The conversion therapy provides a new therapeutic option in clinical practice. Through multidisciplinary treatment (MDT) mode, the patients are given reasonable chemotherapy, radiotherapy and targeted treatment, so that the initial non-resectable tumor can be transformed into resectable tumor, in order to prolong the survival outcome and improve the quality of life of the patients. More and more literatures reported that patients who responded to chemotherapy or targeted treatment and then underwent gastrectomy (stage IV gastric cancer) had longer survival time [3–6].

The combination of oxaliplatin and S-1 (SOX) has been widely used in neoadjuvant and postoperative chemotherapy of gastric cancer [7–9]. Apatinib, a new small molecular inhibitor of vascular endothelial growth factor receptor, which is a third-line treatment option for advanced gastric cancer, has achieved reliable results [10] The combination of SOX and apatinib has also been applied in neoadjuvant chemotherapy of advanced gastric cancer [11]. However, there are few reports on its application in conversion therapy, especially on the survival rate. Therefore, this study examined the short-term survival effect and safety of apatinib combined with oxaliplatin and S-1 in the treatment of unresectable gastric cancer.

## Patients and methods

### Patients

All the patients were confirmed to be adenocarcinoma by biopsy under gastroscopy, and HER-2 negative by immunohistochemistry. Inclusion criteria were: age 18–70 years; confirmed by pathology as gastric adenocarcinoma, and met one of the following non-resectable conditions: peritoneal metastasis, liver metastases, Krukenberg tumor, distant lymph node metastasis, N3, extensive or bulky lymph nodes, local progression; CT/MRI before operation or color ultrasound, PET-CT, if necessary, laparoscopic exploration to clearly diagnose the above-mentioned staging of gastric cancer; diagnosed patients who had not received prior radiotherapy, chemotherapy, targeted treatment or immunotherapy; ECOG 0–1; expected survival time ≥ 3 months; no severe dysfunction of heart, lung and liver; no jaundice and digestive tract obstruction; no acute infection; normal function of main organs; no pregnancy; without involving other clinical trial.

### Treatment

The patients were treated with apatinib, oxaliplatin and S-1. Oxaliplatin was administered every 3 weeks at 130 *mg/m2* intravenously on day 1 according to the body surface area; Apatinib was given orally (500 mg/day) for continuous 5 cycles, followed by a one-cycle rest. S-1: according to the body surface area, the dosage (< 1.25 m^2^, 40 mg, bid; 1.25–1.50 m^2^, 50 mg, bid; > 1.50 m^2^, 60 mg, bid, P. O, bid, d1-d14) was given orally from the first day of chemotherapy for 2 weeks, followed by a 1-week break. The short-term efficacy was evaluated every two cycles. The operation was performed when the requirements of surgical resection were met. All the patients received six cycles of conversion therapy (the last cycle stopped using apatinib). According to RECIST 1.1, complete response (CR) and partial response (PR) were regarded as objective remission rate (ORR), while CR, PR and stable disease (SD) were regarded as disease control rate (DCR). The adverse reactions were divided into 0-IV degrees according to the National Cancer Institute Common Terminology Criteria for Adverse Events (NCI-CTCAE 4.0).

Three weeks after the therapy, the doctors performed conventional laparotomy or laparoscopic exploration, and decided the operation plan based on the situation. D2 radical resection was the first choice. Palliative resection and/or short circuit operation of digestive tract were selected if radical resection of local cancer was not feasible. In the surgical group, the original treatment was continued for two cycles after operation, and then S-1 and apatinib were taken orally for six months. The pathological response of the surgical group was evaluated in accordance with the 14th edition of Japanese gastric cancer treatment protocol, in which the pathological response rate (PRR) refers to the survival of tumor cells in less than two-third of the tumor area (IB or above). Tumor staging (cTNM and ypTNM) was determined according to the AJCC seventh edition.

Each patient was followed-up once every three months after adjuvant treatment until death. The follow-up methods included telephone, short message, outpatient visit, etc. The follow-up items included survival, endoscopy, abdominal enhanced CT, tumor markers and routine laboratory tests.

### Statistical analysis

According to the historical data of SOX conversion therapy, R0 resection rate (H0 value): 36% [7–9]; Expected SOX+Apaninb conversion therapy, R0 resection rate(H1): 60%; bilateralα = 0.05, β = 0.2. The calculated sample size is: *n* = 32, considering the shedding rate of 20%, a total of 40 patients need to be included in the group. SPSS 19.0 statistical software was used for statistical analysis. All measurement data with normal distribution are expressed in (*x ± s*). The mean value of the two groups was compared by the Student’s *t*-test, and the count data were tested by the *χ2* test or the *Fisher* exact probability method. *Kaplan-Meier* method was used for survival analysis, and the survival rate was compared by *Log-rank* test. A *p-value* < 0.05 indicated statistically significant difference*.*

## Results

### Patient characteristics

Between December 2016 and May 2019, 39 patients with advanced unresectable gastric cancer were selected. The average age of the patients was 58 years. The pathological types were signet ring cell carcinoma and poorly differentiated adenocarcinoma. The major non-resectable factors were peritoneal metastasis, N3, liver metastasis and local progression, as shown in Table 1*.*

**Table 1:** Baseline characteristics of the patients

Variable	Patients (***N*** = 39)
Age (years), median (range)	58 (30–68)
Gender, N (%)	
Male	19 (48.7)
Female	20 (51.3)
ECOG performance status, N (%)	
0	12 (30.8)
1	27 (69.2)
Non resectable factors, N (%)	
N3 lymph node metastasis	2 (5.1)
Distant lymphatic metastasis	4 (10.3)
Invasion of peripheral organs (T4b)	4 (10.3)
Liver metastasis	2 (5.1)
Peritoneal carcinomatosis	27 (69.2)
Pathological type, N (%)	
Moderately differentiated adenocarcinoma	5 (12.8)
Poorly differentiated adenocarcinoma	17 (43.6)
Mucinous adenocarcinoma	3 (7.7)
Signet ring cell carcinoma	14 (35.9)
Tumor site, N (%)	
Antrum gastric angle	11 (28.2)
Gastric body	11 (28.2)
Fundus cardia	10 (25.6)
Diffuse whole stomach	7 (17.9)

### Efficacy evaluation

No patient was lost to follow-up. The follow-up time was 12–40 months up to April 2020. After pre-operative treatment, 37 patients could be evaluated (one case was retreated due to IV myelosuppression, and one case was dropped-out due to acute upper gastrointestinal hemorrhage and perforation). Among the 37 patients, one patient achieved CR (2.7%), 26 achieved PR (70.3%), three had SD (8.1%) and seven had PD (18.9%). The ORR was 73.0%, and DCR was 81.1%.

**Fig. 1:**
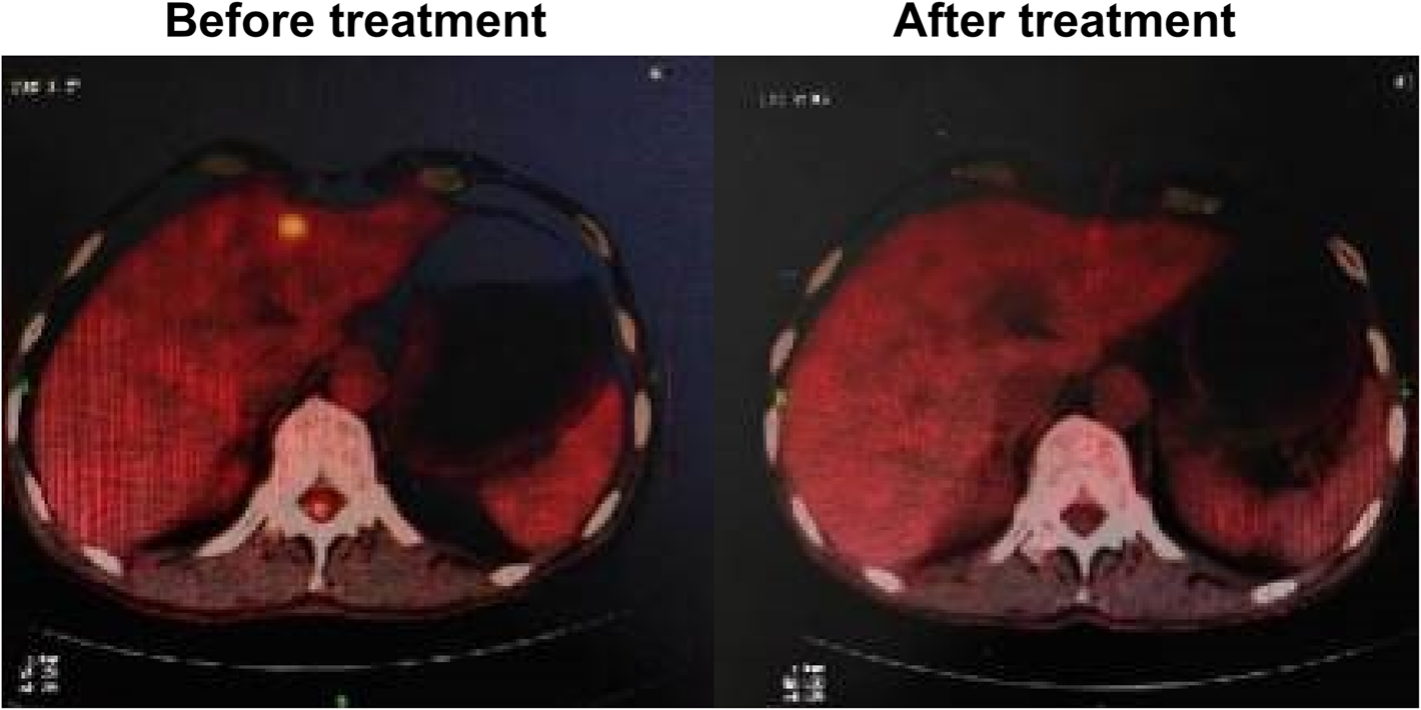
Case 1 (liver metastasis alone)

**Fig. 2:**
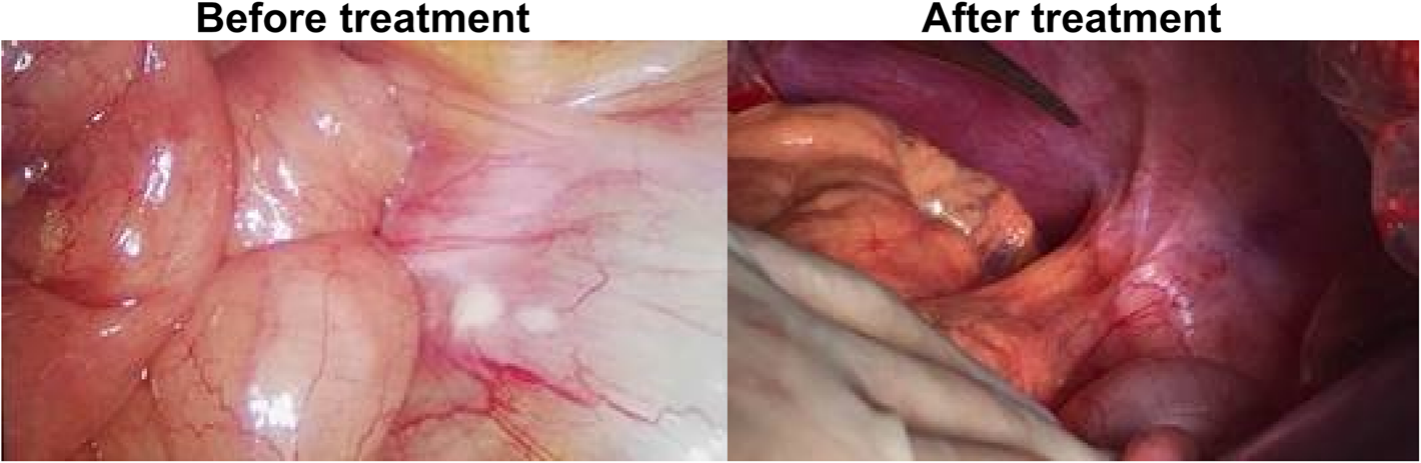
Case 2 (Peritoneal metastasis)

All patients without PD were advised to undergo laparoscopic exploration again and receive surgical treatment as possible.9 patients with PR were not willing to accept surgery due to personal reasons (Table 2).Finally,a total of 22 patients (59.6%) underwent surgery after laparoscopic exploration confirming the curative efficacy, of which 14 underwent radical resection (R0). Intraoperative R0 resection refers to the removal of all visible metastases on the basis of open standard D2 radical surgery, and ensure sufficient margin. The rate of R0 resection was 63.6% (Table 3). PET-CT changes of one case with liver metastasis alone before and after the conversion treatment are shown in Fig. 1. The images of exploration of another case of peritoneal metastasis before and after the conversion treatment are shown in Fig. 2.

**Table 2:** Characteristics of surgical group and non surgical group

Variable	Surgical group, N (%)	Non Surgical group, N (%)
Efficacy evaluation		
CR	1 (4.5)	0 (0)
PR	18 (81.8)	9 (42.9)
SD	3 (13.7)	0 (0)
PD	0 (0)	7 (57.1)
Unresectable factors		
N3 lymph node metastasis	2 (9.1)	0 (0)
Distant lymphatic metastasis	2 (9.1)	2 (12.4)
Invasion of peripheral organs (T4b)	3 (13.7)	1 (6.3)
Liver metastasis	1 (4.5)	1 (6.3)
Peritoneal carcinomatosis	14 (63.6)	12 (75.0)

**Table 3:** Total efficacy evaluation

Variable	Patients, N (%)
CR	1 (2.7)
PR	26 (70.3)
SD	3 (8.1)
PD	7 (18.9)
ORR	27 (73.0)
DCR	30 (81.1)
Surgical conversion	22 (59.5)
R0 resection	14 (63.6)

### Safety and adverse events

The incidence of adverse events (AEs) was 100%. Leucopenia (65.3%), granulocytopenia (69.2%) and thrombocytopenia (15.4%) were the most common hematological AEs. The most common non-hematological AEs included fatigue (51.3%), oral mucositis (35.9%), and hypertension (25.6%). No serious surgical complications were observed (Table 4).

**Table 4:** Incidence of adverse reactions

Variable	Patients (N = 39)	
	Any grade, N (%)	Grade 3 or 4, N (%)
Leukopenia	27 (73.0)	0 (0)
Granulocytopenia	29 (78.4)	0 (0)
Thrombocytopenia	6 (16.2)	1 (2.7)
Elevated transaminase	9 (24.3)	0 (0)
Hand-foot syndrome	9 (24.3)	0 (0)
Stomatitis	14 (37.8)	0 (0)
Fatigue	20 (54.1)	0 (0)
Proteinuria	4 (10.8)	0 (0)
Gastrointestinal hemorrhage	2 (5.4)	1 (2.7)
Hypertension	10 (27.0)	1 (2.7)
Neurotoxicity	10 (27.0)	0 (0)
Gastrointestinal perforation	1 (2.7)	1 (2.7)

### Postoperative pathological examination

In terms of postoperative pathological response, seven cases were Grade IA (31.8%), nine cases were Grade IB (40.9%), five cases were Grade II (22.7%), one case was Grade III (4.5%), and the pathological response rate (PRR) was 68.2% (Table 5).

**Table 5:** Postoperative pathological examination

Variable	Patients, N (%)
Histologic grade (*N* = 22)	
G1. Well differentiated	0 (0)
G2. Moderately differentiated	3 (13.6)
G3. Poorly differentiated	18 (64.3)
G_X_. Not evaluated	1 (4.6)
Pathological response (N = 22)	
Grade 0 (no effect)	0 (0)
Grade I (slight effect)	16 (72.7)
Grade I a (very slight effect)	7 (31.8)
Grade I b (slight effect)	9 (40.9)
Grade II (considerable effect)	5 (22.7)
Grade III (complete response)	1 (4.6)

### Short-term survival efficacy

**Fig. 3:**
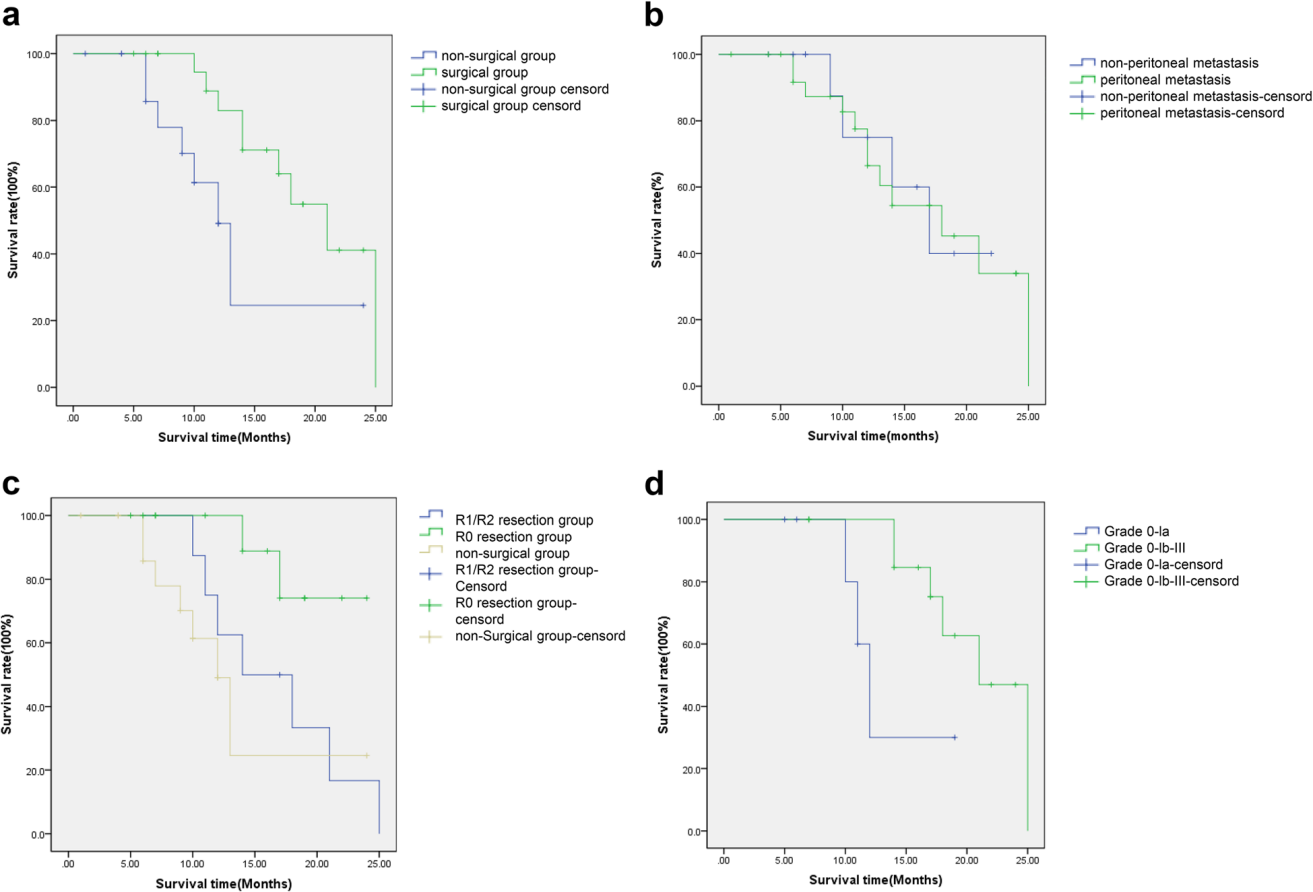
Shor-term survival effect. **a** Overall survival curves of the non-surgical and surgical groups; **b** Overall survival curves of the non-peritoneal metastasis and peritoneal metastasis groups; **c** Overall survival curves of the R1/R2 resection group, R0 group and non-surgical group; **d** Overall survival curves of the different pathological responses

The 1-year survival rate of the surgical group (22 patients) was 71.1%, the 2-year survival rate was 41.1%, and the median survival time was 21 months. The 1-year survival rate of the non-surgical group (15 patients) was 61.4%, the 2-year survival rate was 24.5%, and the median survival time was 12 months. There was a significant difference between the two groups in short-term survival (*p* = 0.026) (Fig. 3a).

Among the 27 patients with peritoneal metastasis, the 1-year survival rate was 77.5%, the 2-year survival rate was 34.0%, and the median survival time was 18 months. Among the 12 patients without peritoneal metastasis, the 1-year survival rate was 75.0%, the 2-year survival rate was 40.0%, and the median survival time was 17 months. There was no significant difference between the two groups in short-term survival (*p* = 0.084) (Fig. 3b).

In the surgical group, 14 cases received R0 resection. Among the 14 patients, 4 patients had recurrence and metastasis during follow-up, 2 patients had peritoneal metastasis, 1 patient had liver metastasis, and 1 patient had bilateral ovarian metastasis. The 1-year survival rate was 88.9%, the 2-year survival rate was 74.1%. 8 cases received R1/R2 resection. The 1-year survival rate was 62.5%, the 2-year survival rate was 0%. There was a significant difference between the two groups in short-term survival (*p* = 0.049), but there was no significant difference between the non-surgical group and the R1/R2 resection group (*p* = 0.186) (Fig. 3c).

In the surgical group, seven cases were diagnosed with pathological grade 0-Ia. The 1-year survival rate was 30.0%. The 2-year survival rate was 0%. The median survival time was 12 months; 15 cases were diagnosed with grade Ib-III. The 1-year survival rate was 84.6%, 2-year survival rate was 47.0%. The median survival time was 21 months. There was significant difference between the two groups in short-term survival (*p* = 0.031) (Fig. 3d).

## Discussion

This was a single arm, phase II clinical study. The main purpose of this study was to evaluate the conversion effect of SOX regimen combined with apatinib in the treatment of unresectable gastric cancer. The primary endpoints were R0 resection rate and safety. The secondary outcome measures were the objective response rate (ORR) and overall survival rate (OSR) of the whole group. Although the follow-up time was < 5 years, we believe that the analysis of short-term survival effect would help to identify the improvement of prognosis of the combined regimen to a certain extent.

In terms of safety, given that the pre-operative course of conversion treatment was up to six cycles,and oral maintenance therapy was needed for half a year after operation. Only three patients (8.1%) in this study had grades III and IV adverse reactions. One patient was withdrew due to grade IV severe thrombocytopenia, one patient withdrew due to emergency operation for upper gastrointestinal hemorrhage and perforation. In the case of repeated grade III hypertension and poor antihypertensive effect, one patient could control the blood pressure level within the normal range after reducing the dosage of apatinib to 250 mg per day, and then received surgical treatment. Apatinib is an anti-angiogenic drug that may induce gastrointestinal perforation and bleeding, which is most likely to cause treatment interruption. However, in this study, except for one patient with upper gastrointestinal hemorrhage and perforation, only one patient had black stool symptoms in the sixth cycle of pre-operative treatment. At that time, the patient had stopped using apatinib for 2 weeks, and all S-1 oral drugs had been consumed, so after fasting, omeprazole and somatostatin treatment, the bleeding stopped quickly. And then the patient successfully underwent R0 resection. Therefore, the combined therapy does not highlight the side effects of apatinib on anti-angiogenesis. The main AEs were myelosuppression, oral mucositis and asthenia. Myelosuppression was mainly leukopenia and granulocytopenia, mostly grade I and II, which were tolerable. Oral mucositis was also mild, and could be relieved after vitamin C treatment and local symptomatic treatment. The incidence of adverse reactions was not higher than that of oxaliplatin and S-1 alone [7, 9]. The incidence of asthenia, hypertension, albuminuria and hand foot syndrome was higher than that of oxaliplatin and S-1 alone reported in the literature [7, 9], but could be easily controlled due to the mild degree of I and II, which did not affect its clinical use. Sym et al. [12]. used docetaxel + capecitabine + cisplatin to treat unresectable gastric cancer, and found that grade 3–4 adverse reactions of preoperative treatment reached 69%. Kinoshita et al. [13]. used docetaxel + cisplatin + S-1 to treat unresectable gastric cancer, and reported that grade 3–4 adverse reactions of preoperative treatment reached 31.6%.In contrast, SOX + apatinib has a relatively mild incidence of grade 3–4 adverse reactions.

The surgical conversion rate of this study is 59.5%.The surgical conversion rate reported in the literature is 59.6–73% [[3, 6, 12, 13]. The surgical conversion rate of this study is not high. The reason is that 9 patients with PR in this study are not willing to accept surgical treatment. Among the patients who underwent R0 resection, one patient achieved pathological complete remission (PCR), which indicated that the scheme had a good conversion effect. No serious complications occurred in any patient during the peri-operative period. The pathological response rate (PRR) reached 68.2%. This standard is widely used in postoperative pathological evaluation of gastric cancer in Asia [14–16]. For the evaluation of primary gastric cancer, the PRR objectively reflects the effectiveness of the combined treatment scheme for patients with advanced gastric cancer.

In terms of survival benefits, the 1-year survival rate of the surgical group was 71.1%, and the 2-year survival rate was 41.1%. The median survival time was 21 months, which was much longer than that of the non-surgical group in this study, and also longer than patients with advanced gastric cancer who received chemotherapy alone reported in the literature [2], suggesting that the successful conversion and concurrent operation can improve the prognosis of the patients. Kinoshita et al. [13]. used docetaxel + cisplatin + S-1 to treat unresectable gastric cancer, and reported that the 1-, and 2-year OS rates were 70.6 and 49.6%, respectively with an MST of 20.9 months. These results suggest that the short-term survival effect of SOX + apatinib in convesion therapy may be close to that of the combination of three chemotherapy drugs. However, there was no significant difference in survival between the peritoneal and non-peritoneal metastasis patients after treatment, suggesting that anti-angiogenesis therapy combined with chemotherapy is also one of the feasible schemes of conversion therapy, even for peritoneal metastasis patients.

The 1-year survival rate and 2-year survival rate of the R0 resection group were 88.9 and 74.1%, respectively, which were similar to the short-term survival of stage III gastric cancer [17–20], which is the ultimate goal of gastric cancer conversion treatment. However, the R1/R2 resection group did not show survival advantage compared with the non-surgical group, which indirectly indicated that if the patients fail to achieve R0 resection after the conversion therapy, surgery may not improve the prognosis. Yamaguchi et al. [21] conducted a multi institution study on 77 patients with stage IV gastric cancer who underwent conversion surgery, and found that the median survival time of patients with R0 was 41.3 months, while that of patients with R1/2 was 21.2 months. This result is similar to that of this study. The 1-year survival rate of patients who achieved PRR was 84.6%, while the 1-year survival rate of patients who failed to achieve PRR was only 30.0%, which means that the PRR is not only an objective index of efficacy evaluation but also an option for prognosis evaluation.

For a single-arm exploratory clinical study, there are many limitations such as the limited samples size, single research site, and single study population. We will also carry out multi center controlled clinical trials to further verify the feasibility of the scheme.

## Conclusions

In summary, apatinib combined with oxaliplatin and S-1 showed a good short-term survival effect and acceptable safety, especially in patients who were able to undergo R0 resection and achieved grade Ib-III of pathological response after resection.

## Data Availability

All data generated or analyzed during this study are included in this published article.

## Abbreviations

R0: Radical resection
CR: Complete response
PR: Partial response
SD: Stable disease
PD: Progressive disease
ORR: Objective response rate
DCR: Disease control rate
AEs: Adverse reactions
MDT: Multidisciplinary treatment
SOX: Oxaliplatin and S-1
PRR: Pathological response rate
OSR: Overall survival rate
PCR: Pathological complete remission
